# Thermal Condensation of Glycine and Alanine on Metal Ferrite Surface: Primitive Peptide Bond Formation Scenario

**DOI:** 10.3390/life7020015

**Published:** 2017-03-27

**Authors:** Md. Asif Iqubal, Rachana Sharma, Sohan Jheeta, Kamaluddin

**Affiliations:** 1 Department of Chemistry, Indian Institute of Technology Roorkee, Roorkee 247 667, Uttarakhand, India; asifiqubal.88@gmail.com (M.A.I.); sharmarachana90@gmail.com (R.S.); 2 Network of Researchers on Horizontal Gene Transfer and Last Universal, Common Ancestor Leeds, Leeds LS7 3RB, UK; sohan@sohanjheeta.com

**Keywords:** amino acids, oligomerization, peptide bond, metal ferrite, surface area

## Abstract

The amino acid condensation reaction on a heterogeneous mineral surface has been regarded as one of the important pathways for peptide bond formation. Keeping this in view, we have studied the oligomerization of the simple amino acids, glycine and alanine, on nickel ferrite (NiFe_2_O_4_), cobalt ferrite (CoFe_2_O_4_), copper ferrite (CuFe_2_O_4_), zinc ferrite (ZnFe_2_O_4_), and manganese ferrite (MnFe_2_O_4_) nanoparticles surfaces, in the temperature range from 50–120 °C for 1–35 days, without applying any wetting/drying cycles. Among the metal ferrites tested for their catalytic activity, NiFe_2_O_4_ produced the highest yield of products by oligomerizing glycine to the trimer level and alanine to the dimer level, whereas MnFe_2_O_4_ was the least efficient catalyst, producing the lowest yield of products, as well as shorter oligomers of amino acids under the same set of experimental conditions. It produced primarily diketopiperazine (Ala) with a trace amount of alanine dimer from alanine condensation, while glycine was oligomerized to the dimer level. The trend in product formation is in accordance with the surface area of the minerals used. A temperature as low as 50 °C can even favor peptide bond formation in the present study, which is important in the sense that the condensation process is highly feasible without any sort of localized heat that may originate from volcanoes or hydrothermal vents. However, at a high temperature of 120 °C, anhydrides of glycine and alanine formation are favored, while the optimum temperature for the highest yield of product formation was found to be 90 °C.

## 1. Introduction

The origin of life can be characterized by an intertwined nexus of biochemical reactions in which a crucial role is being played by proteins. Amino acids are important in biological systems in that they are the building blocks of proteins and thus are composed of long chains of amino acids. Important catalytic activities (e.g., aminoacylase) and the structural integrity (e.g., transmembrane proteins) of cells are manifested by these proteins; such important catalytic activities being performed by aptly named protein molecules called enzymes. The side chains of amino acids in a secondary structure of proteins can interact and bond in any number of possible ways that come to govern the 3-dimensional (3D) structure of proteins. Basically, proteins not only act as a catalyst in biochemical reactions, but they can also be regarded as being instrumental in all cellular processes, and so are the ‘workhorses’ of the cells. In relation to prebiotic chemical evolution, there is a debate over whether metabolism or genetics came first; the main hypothesis which supports metabolism first is the ‘Protein World Hypothesis’ and the one which supports genetics first is the ‘RNA World Hypothesis’. The fundamental opinion of molecular biology is that the replication of nucleic acids was dependent on protein mediated reactions, namely enzymatic and, that the synthesis of these enzymes was dependent on nucleic acids with the result that proteins and nucleic acids are complementary to each other. In contrast, in 1989, the Nobel Prize was awarded to Altman and Cech for their studies on the catalytic activity of RNA [1,2]. In view of their investigation it became apparent that RNA molecules preceded metabolism, because such molecules could carry genetic information and take part as a bio-catalyst as well as having structural integrity. However, several recently published studies have challenged the monopoly of RNA molecules with respect to the RNA world [3,4,5]. Among them, special attention should be paid to the research of Li [4], where they have demonstrated the capability of ancient peptide catalysts towards genetic code formation. The dilemma regarding the question of which came first, proteins (i.e., metabolism) or nucleic acids, remains a matter of great debate for the prebiotic chemistry community to this day, whereas there are some recent theoretical works found in the literature which strive to address this issue [6,7], but establishing the argumentative facts are beyond the scope of this study. The aim of this experimental study is to describe the oligomerization of simple amino acids, namely glycine and alanine, on metal ferrite surfaces, thereby giving more insight with respect to prebiotic peptide formation on mineral surfaces.

The polymerization of amino acids to form primitive protein structures is an indispensable process in view of the chemical evolution of life. However, the condensation of amino acids to form peptides in an aqueous environment is problematic in that it generates water, which is both a thermodynamically and kinetically unfavorable process. Put simply, the free energy associated with a dipeptide bond formation is always higher when compared to that of two individual amino acid molecules, thus inhibiting the equilibrium towards peptide bond formation, resulting in a non-spontaneous process. The solution to this problem may be that if the two amino acids’ overall free energy could be made higher than the free energy of the dipeptide bond in some way, then the direction of the equilibrium of peptide bond synthesis becomes a realistic possibility. Prebiotic chemists ponder as to how peptide bond formation might occur in the conditions found on early Earth. Several approaches have been proposed which attempt to find a solution to this conundrum. Among them, the condensation of amino acids on heterogeneous catalyst surfaces is being considered as one of the widely-accepted frameworks for primitive peptide bond formation. The catalysts in this category are mostly clay minerals [8,9,10,11,12,13,14,15,16,17,18,19,20,21,22,23,24,25,26,27,28,29,30,31,32,33]. However, as metal oxides are recognized as an essential component of the Earth’s crust, the possibility of these compounds acting as heterogeneous catalysts with respect to peptide bond formation cannot be ruled out. The ability of metal oxides and double metal cyanides to promote peptide bond formation has also been studied by other researchers [34,35,36,37,38,39,40,41,42,43,44]. Mineral enhanced hydrothermal oligopeptide formation has also been well demonstrated by Kawamura and co-workers [45].

In order to further explore the role of metal oxides, the metal ferrite nanoparticles, namely nickel ferrite (NiFe_2_O_4_), cobalt ferrite (CoFe_2_O_4_), copper ferrite (CuFe_2_O_4_), zinc ferrite (ZnFe_2_O_4_), and manganese ferrite (MnFe_2_O_4_) were chosen as heterogeneous catalysts to study the oligomerization of the simple amino acids, glycine and alanine, at the temperature range from 50–120 °C for 1–35 days. Metal ferrites are basically binary oxide minerals belonging to the spinel group that have the general formula of A^II^B_2_^III^O_4_, where ‘A’ may be Ni, Co, Cu, Zn, or Mn; and ‘B’ represents Fe. Although several metal oxides have been used in previous oligomerization studies, to the best of our knowledge this important group of binary oxide minerals has not yet been tested. The experimental findings reported here, in relation to the latest use of spinel ferrite for the entrapment of ribonucleotides on its surface [46,47], further sheds light on the role of spinel ferrites during the prebiotic chemistry epoch.

## 2. Experimental Section

### 2.1. Materials and Methods

#### 2.1.1. Materials

Nickel (II) nitrate (Ni(NO_3_)_2_∙6H_2_O), cobalt (II) nitrate (Co(NO_3_)_2_∙6H_2_O), copper (II) nitrate (Cu(NO_3_)_2_∙3H_2_O), citric acid (C_6_H_8_O_7_∙H_2_O) and ethylene glycol (C_2_H_6_O_2_) was purchased from E. Merck. Iron (III) nitrate (Fe(NO_3_)_3_∙9H_2_O), sodium hexane sulphonate, phosphoric acid, acetonitrile, and authentic standard of peptides were purchased from Sigma-Aldrich. The reagents were used without further purification. Millipore water was used throughout the studies.

#### 2.1.2. Preparation of Metal Ferrites

The nanosized metal ferrite, AFe_2_O_4,_ where A = Ni, Co, Cu, and Mn, was prepared in accordance with a previously reported procedure [48]. In a typical synthesis of nickel ferrite, stoichiometric amounts of Ni(NO_3_)_2_∙6H_2_O (0.02 mole, 5.8 g) and Fe(NO_3_)_3_∙9H_2_O (0.04 mole, 16.1 g) were dissolved separately in Millipore water and then mixed together with constant stirring at 80–90 °C. After a complete mixing of the metal salts, citric acid (0.06 mole, 12.6 g) was added to the solution, followed by 10 mL of ethylene glycol. The solution was stirred until gel formation. The obtained gel was subjected to thermal treatment at 400 °C for 2 h in a muffle furnace. This same procedure was used to synthesize the other ferrites.

### 2.2. Characterization of Metal Ferrites

Systematic characterization of the materials has been performed by techniques such as powder X-ray Diffractometry (XRD), Fourier Transform Infrared Spectroscopy (FT-IR), Field Emission Scanning Electron Microscopy (FE-SEM), Transmission Electron Microscopy (TEM), Vibrating Sample Magnetometer (VSM), zero-point charge measurement, surface area analysis, etc. A Brucker AXS D8 Advance XRD was used to characterize the samples. The Perkin-Elmer FT-IR spectrometer was used for acquiring IR spectra of the samples. Surface morphological images as well as the presence of characteristic elements and their atomic ratios were determined by FEI Quanta (FE-SEM) having a 20 kV capacity equipped with an elemental analysis instrument (Energy Dispersive X-ray analysis, EDX). TEM images of the metal ferrite nanoparticles were obtained using a FEI TECHNAI G20 transmission electron microscope operating at 200 kV. The surface area of the samples and their corresponding adsorption/desorption isotherm was determined by a Nova 2200e (Quantachrome) instrument. The specific surface area of the samples was calculated with the help of the Brunauer, Emmet, and Teller (BET) equation [49], which is as follows:

$$\frac{1}{V\left[\left(\frac{P_{0}}{P}\right)-1\right]}=\frac{C-1}{V_{m}C}\left(\frac{P}{P_{0}}\right)+\frac{1}{V_{m}C}$$

where *P*/*P*_0_ represents the relative pressure of nitrogen gas; *V* denotes the amount of *N*_2_ adsorbed; the *N*_2_ adsorbed as the monolayer is *V*_m_; and C is the constant in the BET equation. The specific surface area of the nanoparticles was calculated using the slope and intercept of the linear plot of $1/V\left[\left(\frac{P_{0}}{P}\right)-1\right]$ vs. P/P_0_. Some selected techniques of the material’s characterization have been described briefly in this article. Details of the material’s characterization have been reported in our previous work [46,47].

### 2.3. Oligopeptide Synthesis Protocol from Amino Acid Monomer

The stock solution of the amino acid (glycine and alanine) having a concentration of 0.01 (M) was prepared in a hard glass test tube measuring 150 × 15 mm and an amount of about 0.1 g of each of the pre-weighed metal ferrite solid catalyst was retained. The amino acid solution of 0.1 mL was added to each of the test tubes containing the catalyst, then the suspension obtained was subjected to oven drying for about 3 h at 90 °C. Finally, after the pre-heating treatment, the tube contents were placed in a dry block with three different temperature settings of 50 °C, 90 °C, and 120 °C for 35 days. The progress of the oligomerization reaction was tracked every week with the help of High Performance Liquid Chromatography (HPLC) analysis. To check the catalytic performance of the metal ferrite, a control experiment (without adding a catalyst) was also performed under similar experimental conditions. During the 35-day period, no wetting/drying cycle was deployed. The desorption of amino acids and their oligomeric products was carried out by adding 0.1 M CaCl_2_ solution to each of the test tubes and leaving it to stand for 24 h. The tube contents were filtered through a 0.25 µm syringe filter and the clear filtrate obtained was analyzed by HPLC and Electrospray Ionization Mass Spectrometry (ESI-MS).

### 2.4. High Performance Liquid Chromatography Analysis (HPLC)

HPLC analysis of the reaction mixture was performed via a Waters 2489 binary system with UV detection at 200 nm, using a Purosphere^®^ RP-18 column (250 × 4–6 mm, 5 μm). Sodium hexane sulphonate acidified with phosphoric acid to pH ~ 2.5 (A) and HPLC grade acetonitrile (B) was used as a mobile phase to elute the reaction products. The glycine oligomeric products were eluted with a composition of 100% A, whereas alanine oligomeric products were eluted with a composition of 96% A and 4% B. The mobile phase flow for the HPLC analysis was maintained at 0.50 mL/min in isocratic mode.

### 2.5. Electrospray Ionization-Mass Spectrometry Analysis (ESI-MS)

A Bruker Esquire 4000 (Bruker Daltonic, Bremen, Germany) ion trap mass spectrometer interfaced to an electrospray ionization (ESI) source was used for mass analysis and detection. Ionization of the analytes was carried out using the following setting of the ESI: nebulizer gas flow 10 psi, dry gas 5 L min^−1^, dry temperature 300 °C, capillary voltage 4000 V. Calibration MSn spectra were obtained after isolation of the appropriate precursor ions under similar experimental conditions.

## 3. Results and Discussion

**Figure 1:**
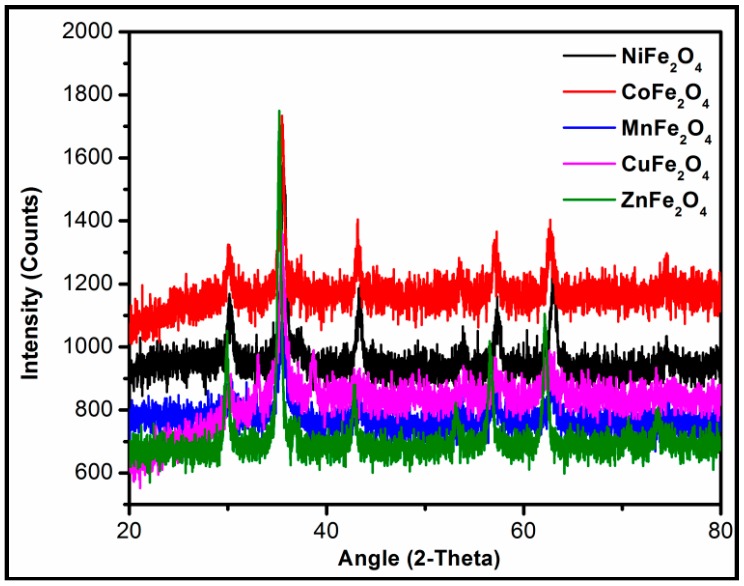
XRD spectra of metal ferrites.

In order to identify the crystal structure and purity of the material, XRD spectra of the samples were taken. Figure 1 shows the XRD spectra of the minerals that are well matched with the Joint Committee on Powder Diffraction Standards (JCPDS) card nos: 00-003-0875, 00-001-1121, 01-077-0010, 10-0319, and 74-2397 for NiFe_2_O_4_, CoFe_2_O_4_, CuFe_2_O_4_, ZnFe_2_O_4_, and MnFe_2_O_4_, respectively, confirming the pure cubic phase spinel structure of the metal ferrites.

**Figure 2:**
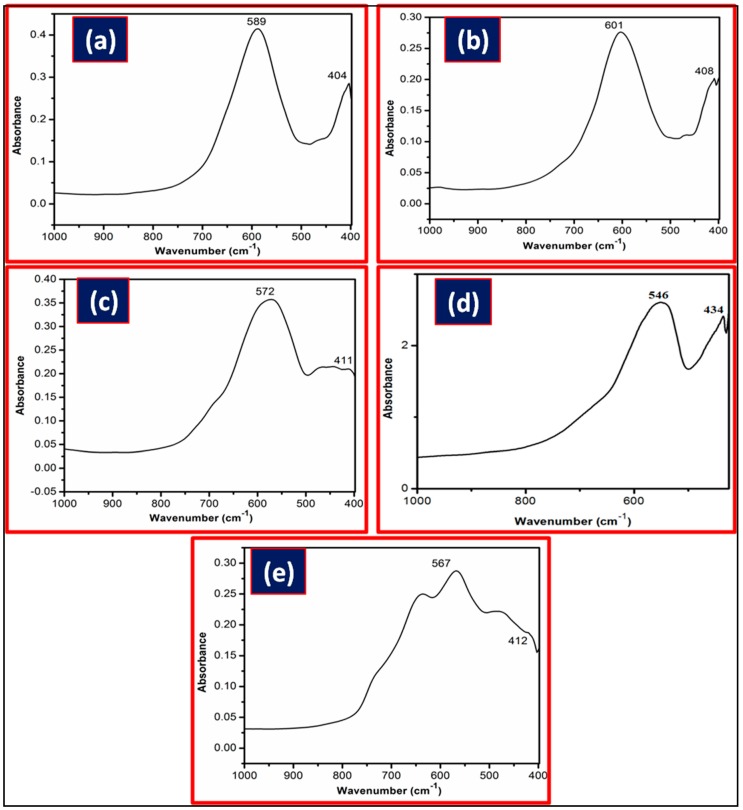
FT-IR spectra of (**a**) NiFe_2_O_4_; (**b**) CoFe_2_O_4_; (**c**) CuFe_2_O_4_; (**d**) ZnFe_2_O_4_; and (**e**) MnFe_2_O_4_.

Characteristic FT-IR peaks of spinel metal ferrite, one in the range of 404–434 cm^−1^ (assigned as metal-oxygen vibration in an octahedral environment) and another between 546 and 601 cm^−1^ (assigned as metal-oxygen vibration in a tetrahedral environment) were observed (Figure 2).

**Figure 3:**
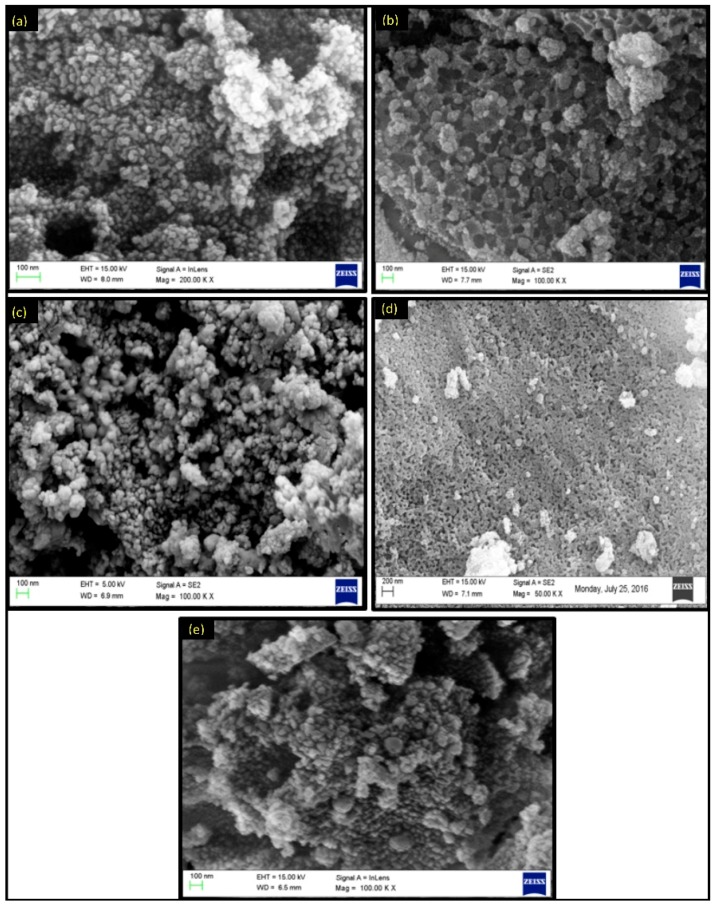
FE-SEM images of (**a**) NiFe_2_O_4_; (**b**) CoFe_2_O_4_; (**c**) CuFe_2_O_4_; (**d**) ZnFe_2_O_4_; and (**e**) MnFe_2_O_4_.

**Figure 4:**
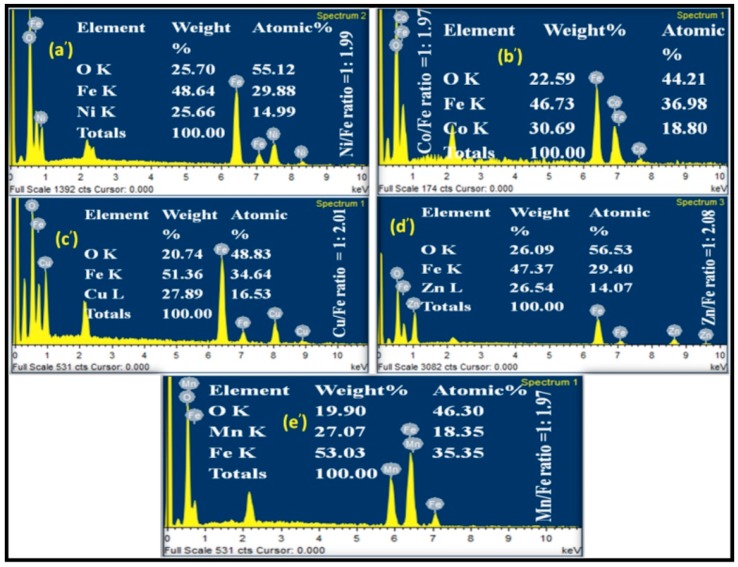
Energy Dispersive X-ray analysis (EDX) spectra of (**a’**) NiFe_2_O_4_; (**b’**) CoFe_2_O_4_; (**c’**) CuFe_2_O_4_; (**d’**) ZnFe_2_O_4_; and (**e’**) MnFe_2_O_4_.

The FE-SEM images shown in Figure 3 revealed that the nanoparticles have an almost spherical structural morphology with a narrow size distribution. Some agglomerated particles are also observed due to high energy generation during the combustion process in the synthesis. The EDX data of the metal ferrite showed that nanoparticles formed without any impurity and the metal and iron ratio (M:Fe) in the synthesized materials is almost close to 1:2 (Figure 4), which is in accord with that of the theoretical ratio.

**Figure 5:**
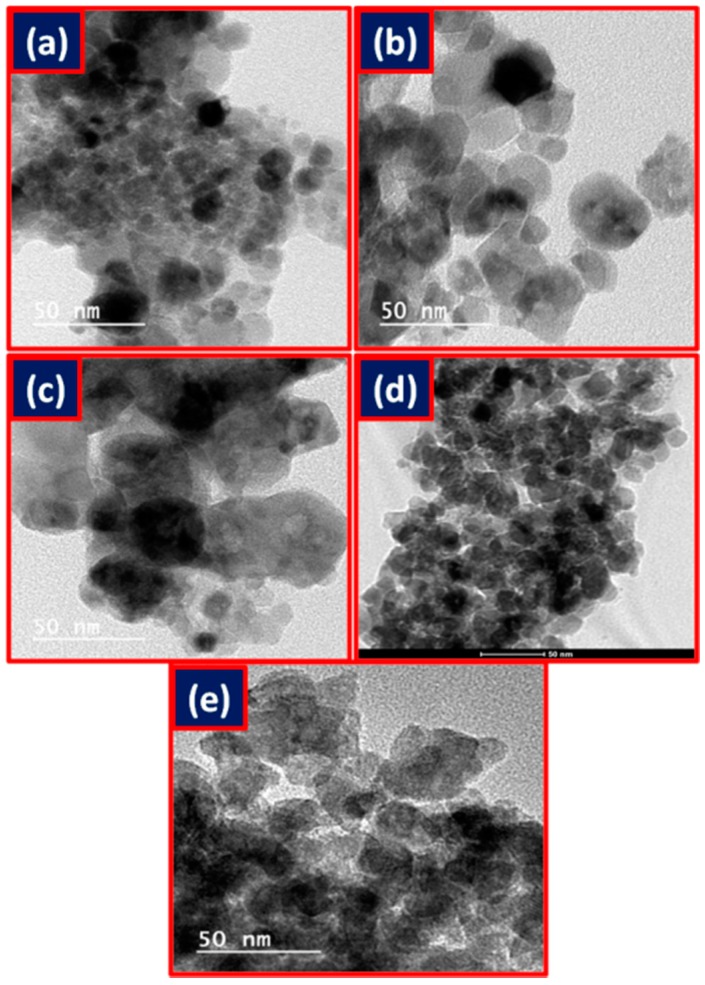
TEM images of (**a**) NiFe_2_O_4_; (**b**) CoFe_2_O_4_; (**c**) CuFe_2_O_4_; (**d**) ZnFe_2_O_4_; and (**e**) MnFe_2_O_4_.

TEM images are given in Figure 5 that also revealed the spherical nature of the nanoparticles. The average particle sizes from the TEM experiment were: 21.7 nm, 20.0 nm, 11.7 nm, 11.4 nm, and 15 nm for NiFe_2_O_4_, CoFe_2_O_4_, CuFe_2_O_4_, MnFe_2_O_4_, and ZnFe_2_O_4_, respectively.

**Figure 6:**
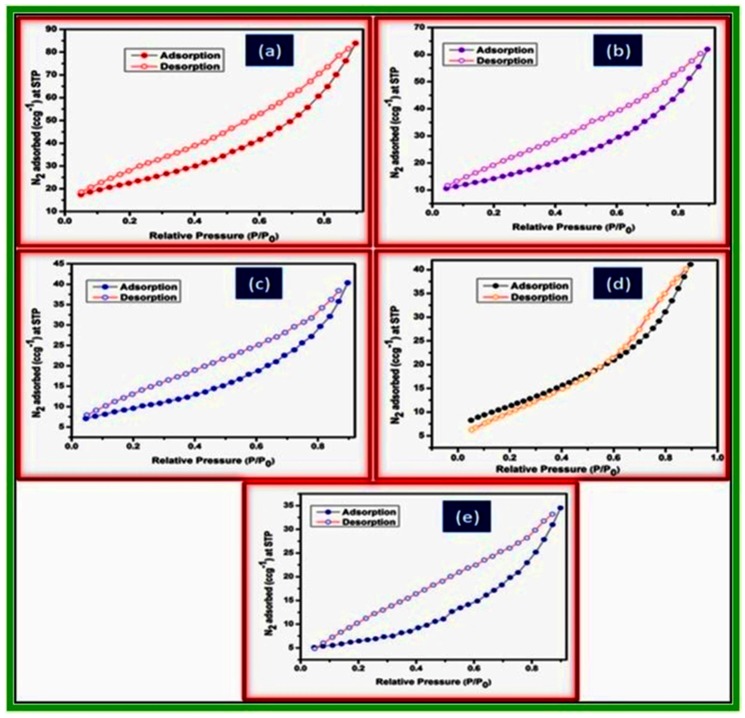
N_2_ adsorption/desorption isotherms of (**a**) NiFe_2_O_4_; (**b**) CoFe_2_O_4_; (**c**) CuFe_2_O_4_; (**d**) ZnFe_2_O_4_; and (**e**) MnFe_2_O_4_.

Figure 6 shows the nitrogen adsorption/desorption isotherms of the metal ferrites. According to the International Union of Pure and Applied Chemistry (IUPAC) classification, all the isotherms can be categorized as type IV isotherm, which is a characteristic of mesoporous materials [50].

The calculated specific surface area of the metal ferrite nanoparticles was in the range of 22.97–80.64 m^2^ g^−1^ (Table 1).

**Figure 7:**
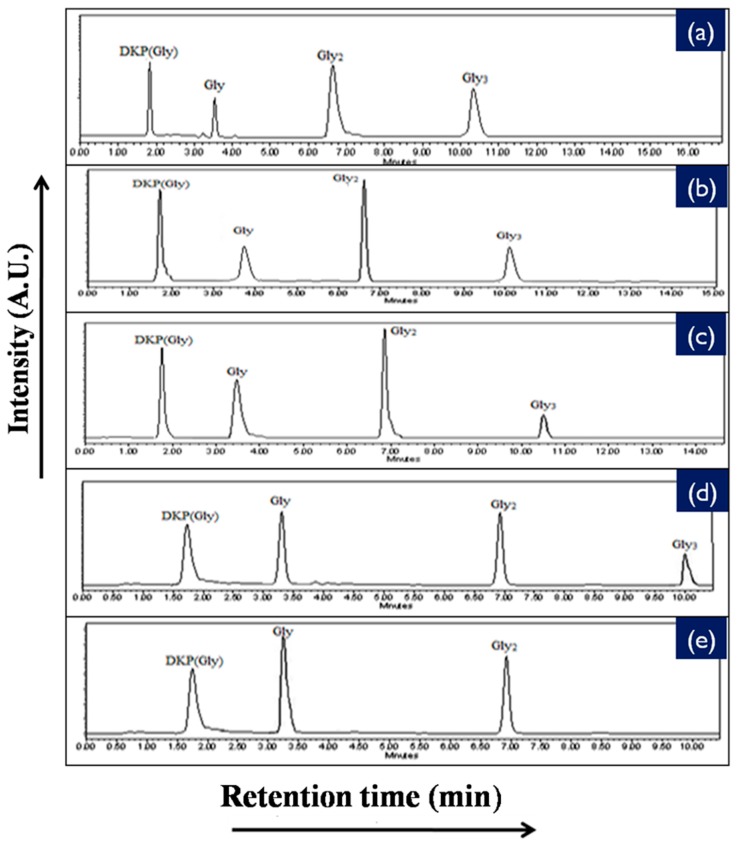
HPLC chromatogram of the products that formed when glycine was heated at 90 °C for 35 days in the presence of (**a**) NiFe_2_O_4_; (**b**) CoFe_2_O_4_; (**c**) CuFe_2_O_4_; (**d**) ZnFe_2_O_4_; and (**e**) MnFe_2_O_4_.

**Figure 8:**
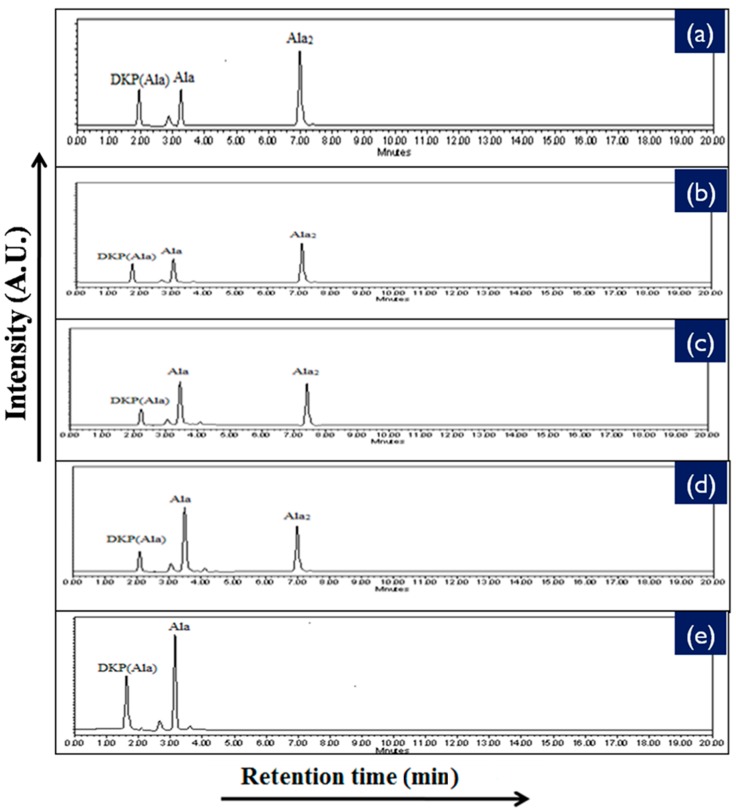
HPLC chromatogram of the products that formed when alanine was heated at 90 °C for 35 days in the presence of (**a**) NiFe_2_O_4_; (**b**) CoFe_2_O_4_; (**c**) CuFe_2_O_4_; (**d**) ZnFe_2_O_4_; and (**e**) MnFe_2_O_4_.

**Figure 9:**
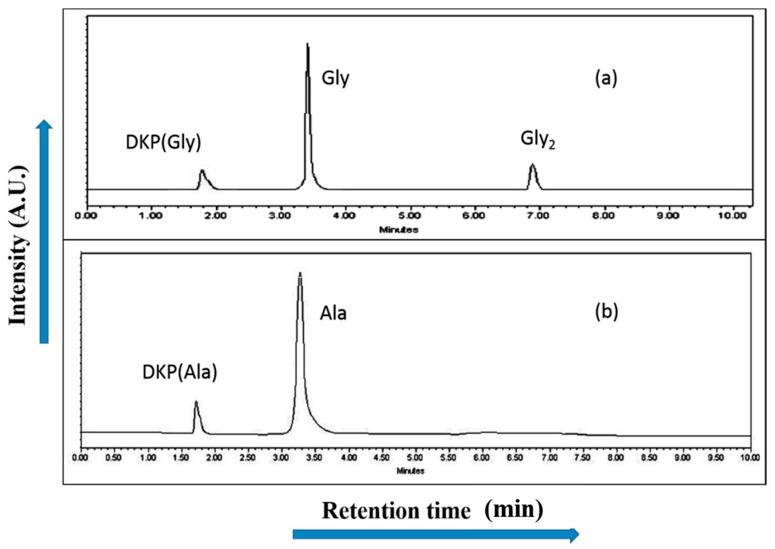
HPLC chromatogram of the products that formed when (**a**) glycine; and (**b**) alanine were heated at 90 °C for 35 days in the control experiment.

To provide further information with respect to the catalyzing properties of the inorganic metal oxides during the oligomerization of the amino acids, the oligomerization reaction of glycine and alanine in the presence of metal ferrite nanoparticles was undertaken at the three temperatures of 50 °C, 90 °C, and 120 °C for durations of 35 days. Formations of oligomeric products were observed on the surface of metal ferrites at different temperatures and times with varying yields. HPLC and ESI-MS techniques were used to identify, as well as quantify, the products obtained in the reaction mixtures. HPLC chromatograms of the reaction products are given in Figure 7 and Figure 8, whilst the control HPLC chromatograms are shown in Figure 9.

The confirmation of the reaction products was carried out by using retention time and the co-injection method. The peak area comparison of products to that of existing standards gave the yield of the reaction products. The yields of the oligomers after 35 days are given in Table 2 and Table 3.

**Figure 10:**
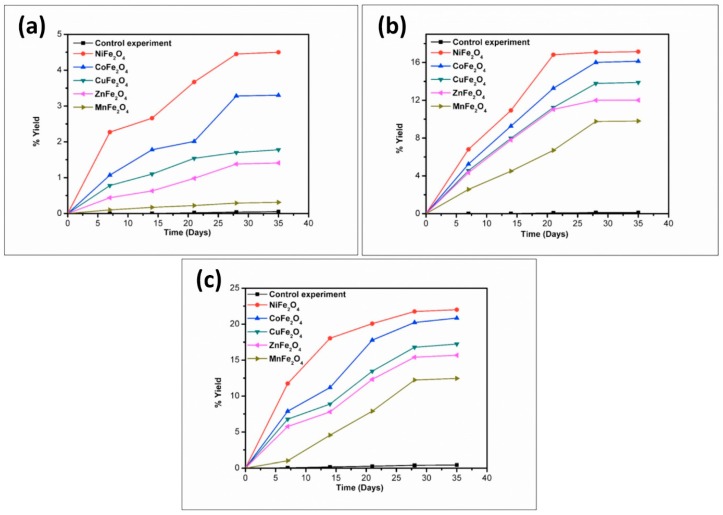
Formation of DKP(Gly) when glycine was heated in the presence of metal ferrites at (**a**) 50 °C; (**b**) 90 °C; and (**c**) 120 °C.

**Figure 11:**
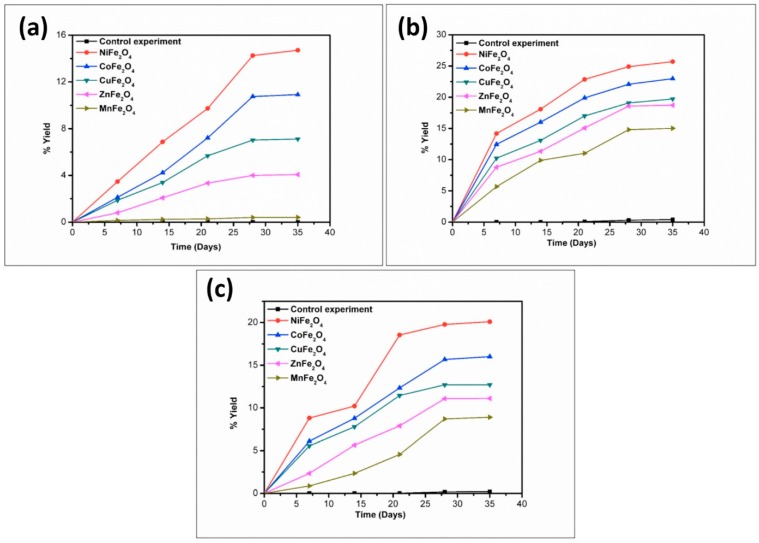
Formation of Gly_2_ when glycine was heated in the presence of metal ferrites at (**a**) 50 °C; (**b**) 90 °C; and (**c**) 120 °C.

**Figure 12:**
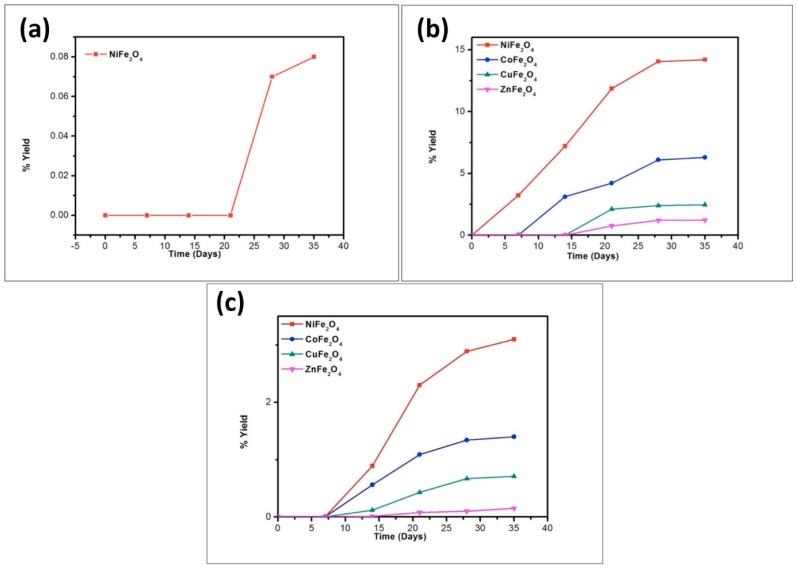
Formation of Gly_3_ when glycine was heated in the presence of metal ferrites at (**a**) 50 °C; (**b**) 90 °C; and (**c**) 120 °C.

**Figure 13:**
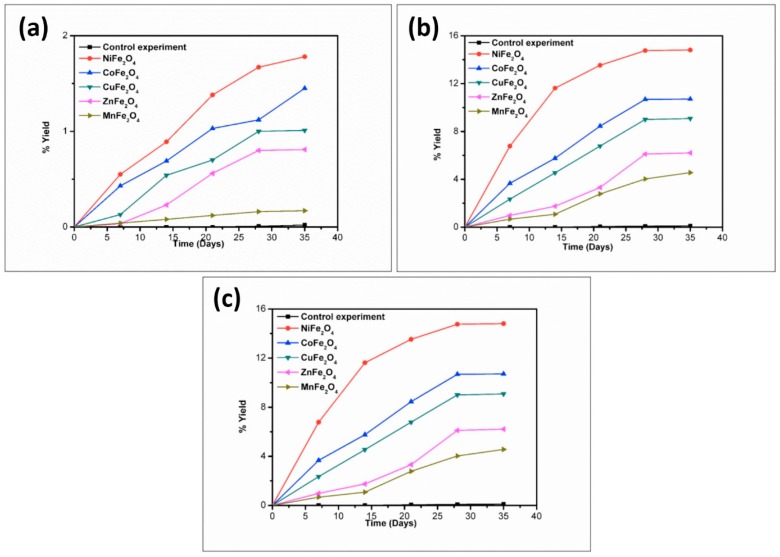
Formation of DKP(Ala) when alanine was heated in the presence of metal ferrites at (**a**) 50 °C; (**b**) 90 °C; and (**c**) 120 °C.

**Figure 14:**
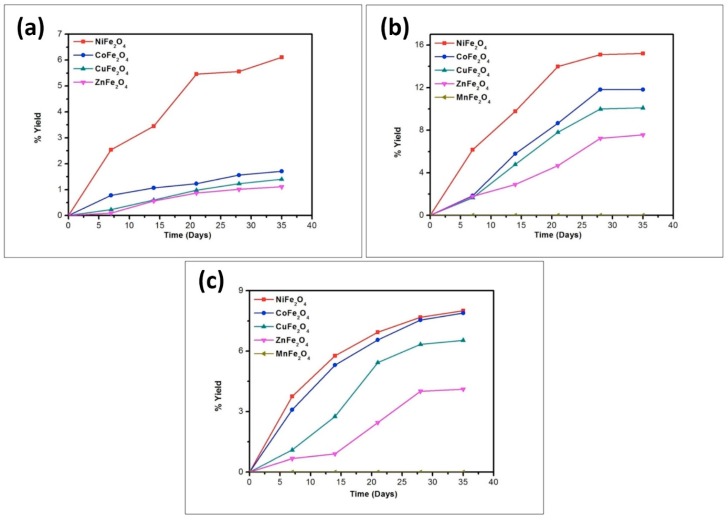
Formation of Ala_2_ when alanine was heated in the presence of metal ferrites at (**a**) 50 °C; (**b**) 90 °C; and (**c**) 120 °C.

To better understand the trend in product formation, the oligomerization reaction was monitored with respect to the parameters of time and temperature. The product formation trend and the yields obtained from the reaction system as a function of temperature and time are given in Figure 10, Figure 11, Figure 12, Figure 13 and Figure 14.

**Figure 15:**
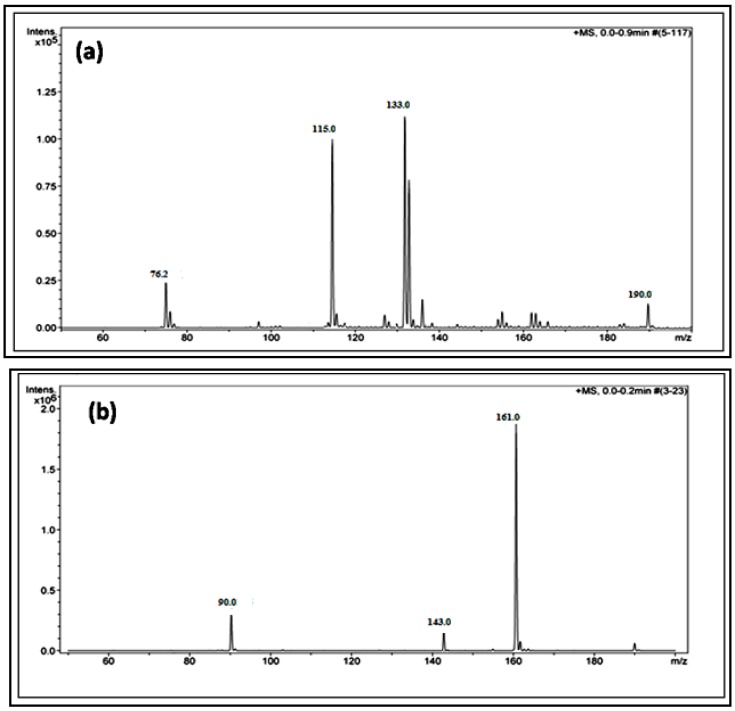
A typical ESI-MS (electrospray ionisation mass spectrometry) spectra of the products that formed when glycine (**a**) and alanine; (**b**) were heated for 35 days at 90 °C in the presence of NiFe_2_O_4_.

The reaction yield against time plot as a function of temperature approximately holds a linear relationship, suggesting the nature of the kinetics is a “pseudo-zero” order one. With the exception of the trimer of glycine, Glycyl-glycyl-glycine (Gly_3_), the glycine and alanine oligomers started to form on the 7th day on each of the catalyst surfaces. The trend during the formation of Gly_3_ with respect to time and temperature varied in relation to all catalysts. The formation of Gly_3_ on NiFe_2_O_4_ at 50 °C was only observed on the 28th day; but at 90 °C, the detection of Gly_3_ was noticed on the 7th day for NiFe_2_O_4_, the 14th for CoFe_2_O_4_, the 21st for CuFe_2_O_4_, and again on the 21st day for ZnFe_2_O_4_. However, at the higher temperature of 120 °C, a time variation for the formation of Gly_3_ was not observed as all the catalysts yielded the product on the 14th day. It is noteworthy to mention here that after analysis of the control experiment (absence of catalyst), only a trace amount of glycine anhydride, herein denoted as DKP(Gly), and a dimer of glycine, Glycyl-glycine (Gly_2_), was identified during the condensation reactions; alanine showed no condensation activity. The result of the control experiment is in agreement with Bujdak and Rode [26,37]. The present metal ferrite catalyzed oligomeric study of glycine demonstrates that glycine can be oligomerized to both dimers and trimers, whereas alanine can be oligomerized to form dimers only. These observations suggest the potential nature of the solid catalyst to initiate condensation of amino acids leading to peptide synthesis at a temperature of less than 100 °C within a short period. Moreover, the formation of peptides at a temperature of 50 °C indicates that the peptide formation process is also possible at low temperatures and did not necessarily need any kind of localized heat arising from volcanoes and hydrothermal vents. As is evident from Figure 7 and Figure 8 as well as Table 2 and Table 3, the peptide synthesis on the surface of metal ferrite nanoparticles may be regarded as a distinctly plausible process which could occur within a relatively short geological time frame. In the case of NiFe_2_O_4_, CoFe_2_O_4_, CuFe_2_O_4_, and ZnFe_2_O_4,_ glycine was oligomerized to a trimer whilst alanine was oligomerized to a dimer with appreciable yield, noting that the formation of Gly_3_ in trace amounts at 50 °C was only observed on the nickel NiFe_2_O_4_ surface. Different levels of dimer synthesis were observed in the case of MnFe_2_O_4_ at 50 °C, but Gly_3_ formation were absent at the higher temperatures: 90 °C and 120 °C (Table 2). Alternatively, MnFe_2_O_4_ gave primarily the cyclic anhydride of alanine, denoted as DKP(Ala), with a trace amount of alanine dimer (Ala_2_) at temperatures of 90 °C and 120 °C. Table 2 and Table 3 show that the yield of the oligomers were found to be higher in the case of glycine compared to alanine. The variation of the yield can be explained by the fact that the formation of alanine peptide bonds needs more energy of activation in comparison to glycine peptide bond formation [15]. Another interesting observation in Table 2 is that the higher the chain length of the amino acids, the lower the yield. The inversely proportional relation between the chain length of the amino acids and the yield of the products is due to the fact that the formation constant of the transition metal-peptide complex is smaller when compared to amino acids [51]. It is important to mention here that synthesis of DKP(Gly) and DKP(Ala) was found to be feasible and was the dominant process at the higher temperature of 120 °C. The high yield of both DKP(Gly) and DKP(Ala) at 120 °C may be explained by the fact that the number of adsorbed water molecules, that is, the thickness of the aqua layer on the surface of metal ferrite, is low in comparison to the surrounding temperature, favoring the dehydration reactions of dimeric glycine and alanine and not chain elongation. That is why, under this condition, the formation of DKP(Gly) and DKP(Ala) from Gly_2_ and Ala_2_ respectively seems to be a more favorable process, compared to that of further chain elongation. The consequence of this could explain the detection of high yields of Gly_3_ at 90 °C rather than at the temperature of 120 °C. Furthermore, some of the characteristic properties of glycine and alanine are also responsible for dominating the condensation reaction by readily producing DKPs. For instance, stereo-chemical feasibility and the short distance between the amino and carboxyl group of alanine molecules facilitates the intramolecular condensation reaction to give DKP(Ala) [52,53,54]. The activation energy of the formation of DKP from Ala_2_ is found to be lower when compared to the formation of Ala_2_ from the condensation of two alanine molecules [55]. The high temperature favoring the formation of DKP in this study is in accord with the following studies [39,42,44,56]. Under hydrothermal conditions, kinetic analysis of dimer formation and DKPs was also reported by Kawamura and co-workers [57,58]. The ESI-MS instrument offers an alternative analytical technique for the identification of the reaction products by producing the mass of the compounds in terms of m/z = (M + H)^+^ ions, where M stands for the analyte (amino acid/oligomer) to be detected. Figure 15 shows the ESI-MS data of the oligomeric product formation relating to glycine and alanine at 90 °C for 35 days. The m/z values 115.0488, 133.0592, 190.0800, 143.0500, and 161.0659 in the mass spectra show the presence of DKP(Gly), Gly_2_, Gly_3_, DKP(Ala), and Ala_2_, respectively, in the reaction mixture. Beside these peaks, one more additional peak in both the spectra (Figure 15a,b) was characterized as monomeric glycine and alanine at m/z 76.2866 and 90.0176, respectively. ESI-MS data gave the results which are in accordance with the results obtained by HPLC.

Among the metal ferrites studied, NiFe_2_O_4_ was found to be the most efficient catalyst for the oligomerization of glycine and alanine because it not only gave the highest yield of oligomeric products, it also showed a trace amount of Gly_3_ even with a temperature as low as 50 °C. MnFe_2_O_4_ was the least efficient catalyst as it produced the lowest yield, as well as shorter oligomers of amino acids under the same set of experimental conditions. It produced only Gly_2_ on its surface and with alanine the major product formed is DKP(Ala), along with a miniscule amount of Ala_2_ at higher temperatures. All the metal ferrites except MnFe_2_O_4_ exhibited low temperature oligomer synthesis activity in terms of Ala_2_ formation at 50 °C, suggesting that MnFe_2_O_4_ has the lowest active catalytic properties of the metal ferrites. The overall trend in the yield of the oligomeric products obtained is as follows:

NiFe_2_O_4_ > CoFe_2_O_4_ > CuFe_2_O_4_ > ZnFe_2_O_4_ > MnFe_2_O_4_

As evident from the surface area data (Table 1) and yield of the products (Table 2 and Table 3), it seems that surface area plays an important role towards the trend in product formation. NiFe_2_O_4_, with the highest surface area (80.64 m^2^ g^−1^) appeared to be the best catalyst, whereas MnFe_2_O_4_ with the lowest surface area of (22.97 m^2^ g^−1^) was found to be least effective. The most plausible explanation for oligomeric product formation on the surface of the metal ferrites seems to be the surface area and surface active hydroxyl groups. The catalytic activity of the metal ferrites arises primarily from the surface-active hydroxyl groups located on the metal ferrite. Chemically highly potent free hydroxyl groups in the aqueous environment on the surface of the metal ferrite played a key role during the condensation reaction by forming intermolecular hydrogen bonds. Moreover, they were exposed directly towards the interacting molecules, leading to better interaction between the metal ferrite and amino acid molecules. It is natural that a degree of interaction between the metal ferrite and amino acids will increase proportionally with the increase of hydroxyl groups available on metal ferrite surfaces, and consequently more products will be yielded. Considering the relationship between the number of free surface reactive hydroxyl groups on the metal ferrite and the surface area of the material, it can be said that the reactivity of these directly exposed hydroxyl groups is directly proportional to the material’s available surface area. Taking these facts together, it can be said that the higher the surface area of the materials, the more hydroxyl groups will be available and consequently the product formation rate will be higher, e.g., NiFe_2_O_4_, having the highest surface area with a maximum number of hydroxyl groups on its surface, has more interaction with amino acids, when compared to other ferrites, thereby producing the highest oligomeric yield.

As discussed earlier, metal ferrites are a group of spinel minerals having the general formula A^II^B_2_^III^O_4_ where ‘A’ may be Zn, Ni, Co, Cu, or Mn and B equals Fe. Special emphasis has been given to these ferrites of metals due to their catalytic activities having some pre-biological significance. Transition metal ions present in the primordial oceans as well as in the Earth’s crust undoubtedly became part of the evolutionary chemistry of life and thus became a vital part of living cells [59]. These spinel type metal ferrite minerals are of special interest because not only are they present in the Earth’s crust [60,61]; they can also be found in meteorites [62,63,64]; hydrothermal vent systems [65]; and ancient sediments [66,67], as well as the sea bed [68,69]. The main objective of this paper is to ascertain the suitability of these specific oxides with respect to the condensation of amino acids and their relevance to the chemical evolution of life.

## 4. Implications for Chemical Evolution

This study demonstrates that metal ferrite nanoparticles of nickel, cobalt, copper, zinc, and manganese catalyzed oligomerization of simple amino acids such as glycine and alanine at temperatures between 50 and 120 °C for periods of between 1 and 35 days. Thus, these nanoparticles may be thought of as prospective heterogeneous catalysts for amino acid condensation reactions in relation to primitive peptide formation during the chemical evolution processes of life. An important finding of this study is the formation of peptides at temperatures as low as 50 °C, indicating that the condensation process is possible at low temperatures and independent of any other type of localized heat source such as volcanoes or hydrothermal vents. What is the significance of these findings in relation to chemical evolution? The presence of these minerals in so wide that a variety of places on Earth render them a vital crystalline structure upon which the sorption and oligomerization of amino acids might have taken place on early Earth, some 4 billion years ago. This would give some credence to the metabolism first hypothesis as opposed to the genetic first one; in that it has been shown that it is possible to synthesize oligomers.

## Figures and Tables

**Table 1:** Surface area of metal ferrites.

Compound	BET (Brunauer–Emmett–Teller) Surface Area (m^2^/g)
NiFe_2_O_4_	80.64
CoFe_2_O_4_	53.66
CuFe_2_O_4_	34.67
ZnFe_2_O_4_	28.54
MnFe_2_O_4_	22.97

**Table 2:** The reaction yield (%) of the products that formed from glycine at temperatures of 50°, 90°, and 120 °C for 35 days in the presence of metal ferrites.

Catalyst	% Yield of the Products Formed When Glycine Was Heated at 50, 90, and 120 °C for 35 Days								
	DKP (Diketopiperazine) (Gly)			(Gly)_2_			(Gly)_3_		
	50 °C	90 °C	120 °C	50 °C	90 °C	120 °C	50 °C	90 °C	120 °C
No catalyst	0.05	0.12	0.43	n.d	0.40	0.20	n.d	n.d	n.d
NiFe_2_O_4_	4.50	17.15	22.01	14.71	25.70	20.10	trace	14.2	3.10
CoFe_2_O_4_	3.30	16.13	20.84	10.91	22.98	16.01	* n.d	6.30	1.40
CuFe_2_O_4_	1.78	13.89	17.23	7.11	19.72	12.71	n.d	2.47	0.71
ZnFe_2_O_4_	1.41	12.01	15.67	4.07	18.70	11.11	n.d	1.23	0.15
MnFe_2_O_4_	0.31	9.80	12.45	0.41	15.01	8.90	n.d	n.d	n.d

* n.d = not detected.

**Table 3:** The reaction yield (%) of the products that formed from alanine at temperatures of 50, 90, and 120 °C for 35 days in the presence of metal ferrites.

Catalyst	% Yield of the Products Formed When Alanine Was Heated at 50, 90 and 120 °C for 35 Days					
	DKP(Ala)			(Ala)_2_		
	50 °C	90 °C	120 °C	50 °C	90 °C	120 °C
No catalyst	0.02	0.07	0.11	n.d	n.d	n.d
NiFe_2_O_4_	1.78	13.11	14.81	6.11	15.21	8.01
CoFe_2_O_4_	1.45	9.23	10.71	1.71	11.81	7.89
CuFe_2_O_4_	1.01	8.81	9.08	1.40	10.1	6.54
ZnFe_2_O_4_	0.81	4.78	6.21	1.11	7.57	4.11
MnFe_2_O_4_	0.17	3.89	4.56	* n.d	trace	trace

* n.d = not detected.

## References

[1] Altman S. (1990). Enzymatic cleavage of RNA by RNA (Nobel lecture). Angew. Chem. Int. Ed..

[2] Cech T.R. (1990). Self-splicing and Enzymatic Activity of an Intervening Sequence RNA from Tetrahymena (Nobel Lecture). Angew. Chem. Int. Ed..

[3] Bernhardt H.S. (2012). The RNA world hypothesis: The worst theory of the early evolution of life (except for all the others). Biol. Direct..

[4] Li L., Francklyn C., Carter C.W. (2013). Aminoacylatingurzymes challenge the RNA world hypothesis. J. Biol. Chem..

[5] Bowman J.C., Hud N.V., Williams L.D. (2015). The ribosome challenge to the RNA world. J. Mol. Evolut..

[6] Kawamura K. (2016). A Hypothesis: Life Initiated from Two Genes, as Deduced from the RNA World Hypothesis and the Characteristics of Life-Like Systems. Life.

[7] Kawamura K. (2012). Drawbacks of the ancient RNA-based life-like system under primitive earth conditions. Biochimie.

[8] Degens E.T., Matheja J., Jackson T.A. (1970). Template catalysis: Asymmetric polymerization of amino-acids on clay minerals. Nature.

[9] Paecht-Horowitz M. (1973). Inorganic clays as possible prebiotic peptide templates. Isr. J. Chem..

[10] Warden J.T., McCullough J.J., Lemmon R.M., Calvin M. (1974). A re-examination of the zeolite-promoted, clay-mediated peptide synthesis. J. Mol. Evolut..

[11] Paecht-Horowitz M. (1976). Clays as possible catalysts for peptide formation in the prebiotic era. Orig. Life Evolut. Biosph..

[12] Paecht-Horowitz M., Lahav N. (1977). Polymerization of alanine in the presence of a non-swelling montmorillonite. J. Mol. Evolut..

[13] Flegmann A.W., Scholefield D. (1978). Thermodynamics of peptide bond formation at clay mineral surfaces. J. Mol. Evolut..

[14] Lahav N., White D., Chang S. (1978). Peptide formation in the Prebiotic era: Thermal condensation of glycine in fluctuating clay environments. Science.

[15] Lawless J.G., Levi N. (1979). The role of metal ions in chemical evolution: Polymerization of alanine and glycine in a cation-exchanged clay environment. J. Mol. Evolut..

[16] Lahav N., White D.H. (1980). A possible role of fluctuating clay-water systems in the production of ordered prebiotic oligomers. J. Mol. Evolut..

[17] Luke B.T., Gupta A.G., Loew G.H., Lawless J.G., White D.H. (1984). Theoretical investigation of the role of clay edges in prebiotic peptide bond formation. I. Structures of acetic acid, glycine, H_2_SO_4_, H_3_PO_4_, Si(OH)_4_, and Al(OH)_4_. Int. J. Quantum Chem..

[18] White D.H., Kennedy R.M., Macklin J. (1984). Acyl silicates and acyl aluminates as activated intermediates in peptide formation on clays. Orig. Life Evolut. Biosph..

[19] Collins J.R., Loew G.H., Luke B.T., White D.H. (1988). Theoretical investigation of the role of clay edges in prebiotic peptide bond formation. Orig. Life Evolut. Biosph..

[20] Bujdak J., Slosiarikova H., Texler N., Schwendinger M., Rode B.M. (1994). On the possible role of montmorillonites in prebiotic peptide formation. Monatsh. Für. Chem..

[21] Bujdák J., Faybíková K., Eder A., Yongyai Y., Rode B.M. (1995). Peptide chain elongation: A possible role of montmorillonite in prebiotic synthesis of protein precursors. Orig. Life Evol. Biosph..

[22] Bujdák J., Le Son H., Yongyai Y., Rode B.M. (1996). The effect of reaction conditions on montmorillonite-catalysed peptide formation. Catal. Lett..

[23] Bujdak J., Rode B.M. (1996). The effect of smectite composition on the catalysis of peptide bond formation. J. Mol. Evolut..

[24] Zamaraev K.I., Romannikov V.N., Salganik R.I., Wlassoff W.A., Khramtsov V.V. (1997). Modelling of the prebiotic synthesis of oligopeptides: Silicate catalysts help to overcome the critical stage. Orig. Life Evolut. Biosph..

[25] Porter T.L., Eastman M.P., Hagerman M.E., Price L., Shand R.F. (1998). Site-specific Prebiotic Oligomerization reactions of Glycine on the surface of Hectorite. J. Mol. Evolut..

[26] Bujdak J., Rode B.M. (1999). The effect of clay structure on peptide bond formation catalysis. J. Mol. Catal. A.

[27] Porter T.L., Eastman M.P., Bain E., Begay S. (2001). Analysis of peptides synthesized in the presence of SAz-1 montmorillonite and Cu 2+ exchanged hectorite. Biophys. Chem..

[28] Aquino A.J., Tunega D., Gerzabek M.H., Lischka H. (2004). Modeling catalytic effects of clay mineral surfaces on peptide bond formation. J. Phys. Chem. B.

[29] Bujdak J., Rode B.M. (2004). On the mechanisms of oligopeptide reactions in solution and clay dispersion. J. Pept. Sci..

[30] Rimola A., Sodupe M., Ugliengo P. (2007). Aluminosilicate surfaces as promoters for peptide bond formation: An assessment of Bernal’s hypothesis by AB Initio methods. J. Am. Chem. Soc..

[31] Pant C.K., Lata H., Pathak H.D., Mehata M.S. (2009). Heat-initiated prebiotic formation of peptides from glycine/aspartic acid and glycine/valine in aqueous environment and clay suspension. Int. J. Astrobiol..

[32] Jaber M., Georgelin T., Bazzi H., Costa-Torro F., Lambert J.F., Bolbach G., Clodic G. (2014). Selectivities in adsorption and peptidic condensation in the (arginine and glutamic acid)/montmorillonite clay system. J. Phys. Chem. C.

[33] Fuchida S., Masuda H., Shinoda K. (2014). Peptide formation mechanism on montmorillonite under thermal conditions. Orig. Life Evolut. Biosph..

[34] Bujdak J., Rode B.M. (1997). Silica, alumina, and clay-catalyzed alanine peptide bond formation. J. Mol. Evolut..

[35] Bujdak J., Rode B.M. (1997). Glycine oligomerization on silica and alumina. React. Kinet. Catal. Lett..

[36] Smith J.V. (1998). Biochemical evolution. I. Polymerization on internal, Organophilic silica surfaces of Dealuminated Zeolites and Feldspars. Proc. Natl. Acad. Sci. USA.

[37] Bujdak J., Rode B.M. (1999). Silica, alumina and clay catalyzed peptide bond formation: Enhanced efficiency of alumina catalyst. Orig. Life Evolut. Biosph..

[38] Bujdak J., Rode B.M. (2001). Activated alumina as an energy source for peptide bond formation: Consequences for mineral-mediated prebiotic processes. Amino Acids.

[39] Basiuk V.A., Sainz-Rojas J. (2001). Catalysis of peptide formation by inorganic oxides: High efficiency of alumina under mild conditions on the earth-like planets. Adv. Space Res..

[40] Bujdak J., Rode B.M. (2002). Preferential amino acid sequences in alumina-catalyzed peptide bond formation. J. Inorg. Biochem..

[41] Matrajt G., Blanot D. (2004). Properties of synthetic ferrihydrite as an amino acid adsorbent and a promoter of peptide bond formation. Amino Acids.

[42] Shanker U., Bhushan B., Bhattacharjee G., Kamaluddin (2012). Oligomerization of glycine and alanine catalyzed by iron oxides: Implications for prebiotic chemistry. Orig. Life Evolut. Biosph..

[43] Guo C., Holland G.P. (2015). Alanine Adsorption and Thermal Condensation at the Interface of Fumed Silica Nanoparticles: A Solid-State NMR Investigation. J. Phys. Chem. C.

[44] Kumar A., Kamaluddin (2012). Oligomerization of glycine and alanine on metal (II) octacynaomolybdate (IV): Role of double metal cyanides in prebiotic chemistry. Amino Acids.

[45] Kawamura K., Takeya H., Kushibe T., Koizumi Y. (2011). Mineral-enhanced hydrothermal oligopeptide formation at the second time scale. Astrobiology.

[46] Iqubal M.A., Sharma R., Kamaluddin (2015). Studies on interaction of ribonucleotides with zinc ferrite nanoparticles using spectroscopic and microscopic techniques. Karbala Int. J. Mod. Sci..

[47] Iqubal M.A., Sharma R., Kamaluddin (2016). Surface Interaction of Ribonucleic Acid Constituents with Spinel Ferrite Nanoparticles: A Prebiotic Chemistry Experiment. RSC Adv..

[48] Sharma R., Bansal S., Singhal S. (2015). Tailoring the photo-Fenton activity of spinel ferrites (MFe_2_O_4_) by incorporating different cations (M = Cu, Zn, Ni and Co) in the structure. RSC Adv..

[49] Brunauer S., Emmett P.H., Teller E. (1938). Adsorption of gases in multimolecular layers. J. Am. Chem. Soc..

[50] Brunauer S., Deming L.S., Deming W.E., Teller E. (1940). On a theory of the van der Waals adsorption of gases. J. Am. Chem. Soc..

[51] Greenstein J.P., Winitz M. (1961). Chemistry of the Amino Acids.

[52] Orgel L.E. (1989). The origin of polynucleotide-directed protein synthesis. J. Mol. Evolut..

[53] Nagayama M., Takaoka O., Inomata K., Yamagata Y. (1990). Diketopiperazine-mediated peptide formation in aqueous solution. Orig. Life Evolut. Biosph..

[54] Rode B.M., Schwendinger M.G. (1990). Copper-catalyzed amino acid condensation in water—A simple possible way of prebiotic peptide formation. Orig. Life Evolut. Biosph..

[55] Cox J.S., Seward T.M. (2007). The reaction kinetics of alanine and glycine under hydrothermal conditions. Geochim. Cosmochim. Acta.

[56] Wu J., Zhang Z., Yu X., Pan H., Jiang W., Xu X., Tang R. (2011). Mechanism of promoted dipeptide formation on hydroxyapatite crystal surfaces. Chin. Sci. Bull..

[57] Kawamura K., Nishi T., Sakiyama T. (2005). Consecutive elongation of alanine oligopeptides at the second time range under hydrothermal conditions using a microflow reactor system. J. Am. Chem. Soc..

[58] Kawamura K., Yukioka M. (2001). Kinetics of the racemization of amino acids at 225–275 °C using a real-time monitoring method of hydrothermal reactions. Thermochimicaacta.

[59] Baudisch O.S. (1943). The importance of trace elements in biologic activity. Am. Sci..

[60] Clarke F.W., Washington H.S. (1924). The Composition of the Earth’s Crust.

[61] Reichmann H.J., Jacobsen S.D. (2006). Sound velocities and elastic constants of ZnAl_2_O_4_ spinel and implication for spinel-elasticity systematics. Am. Miner..

[62] Hoover R.B. Microfossils of cyanobacteria in carbonaceous meteorites. Proceedings of the SPIE 6694, Instruments, Methods, and Missions for Astrobiology X, 669408.

[63] Karwowski L. (2012). Sołtmany meteorite. Meteorites.

[64] Bindi L., Yao N., Lin C., Hollister L.S., Andronicos C.L., Distler V.V., Eddy M.P., Kostin A., Kryachko V., MacPherson G.J. (2015). Natural quasicrystal with decagonal symmetry. Sci. Rep..

[65] Rychagov S.N., Glavatskikh S.F., Sandimirova E.I. (1996). Ore and silicate magnetic pellets as indicators of structure and fluid regime, as well as mineral and ore formation in the present-day Baranskii hydrothermal system, Iturup Island. Geol. Ore Deposits.

[66] Essene E.J., Peacor D.R. (1983). Crystal chemistry and petrology of coexisting galaxite and jacobsite and other spinel solutions and solvi. Am. Miner..

[67] Stalder M., Rozendaal A. (2005). Calderite-rich garnet and franklinite-rich spinel in amphibolite-facies hydrothermal sediments, Gamsberg Zn-Pb deposit, Namaqua Province, South Africa. Can. Miner..

[68] Exon N.F., Raven M.D., Carlo E.D. (2002). Ferromanganese nodules and crusts from the Christmas Island region, Indian Ocean. Mar. Georesour. Geotechnol..

[69] Timofeeva Y.O., Karabtsov A.A., Semal V.A., Burdukovskii M.L., Bondarchuk N. (2014). Iron-Manganese Nodules in Updates: The Dependences of the accumulation of trace elements on nodule size. Soil Sci. Soc. Am. J..

